# (CTG)n repeat-mediated dysregulation of *MBNL1* and *MBNL2* expression during myogenesis in DM1 occurs already at the myoblast stage

**DOI:** 10.1371/journal.pone.0217317

**Published:** 2019-05-22

**Authors:** Laurène M. André, Remco T. P. van Cruchten, Marieke Willemse, Derick G. Wansink

**Affiliations:** Radboud Institute for Molecular Life Sciences, Radboud University Medical Center, Department of Cell Biology, Nijmegen, The Netherlands; University of Minnesota Medical School, UNITED STATES

## Abstract

Myotonic dystrophy type 1 (DM1) is a severe neuromuscular disorder caused by the expression of trinucleotide repeat-containing *DMPK* transcripts. Abnormally expanded (CUG)n repeats in these transcripts form hairpin-like structures that cause the RNA to accumulate in the cell nucleus by sequestering isoforms of the Muscleblind (MBNL) family, tissue-specific regulators of developmentally programmed, post-transcriptional processes in RNA metabolism. Through this mechanism, the function of *MBNL* in RNA processing becomes dominantly perturbed, which eventually leads to aberrant alternative splicing and the expression of foetal splice variants of a wide variety of proteins, including the MBNL isoforms themselves. Here, we employ a patient-derived muscle cell model for DM1 to examine in detail the expression of *MBNL* RNA and protein variants during myogenic differentiation. This DM1 model consists of a panel of isogenic myoblast cell lines that either contain a pathogenic *DMPK* allele with a congenital mutation of 2600 triplets, or lack this expanded repeat through CRISPR/Cas9-mediated gene editing. We found that the temporal expression levels of *MBNL1*, *MBNL2* and *MBNL3* RNAs are not influenced by presence of the (CTG)2600 repeat during myogenesis *in vitro*. However, throughout myoblast proliferation and differentiation to myotubes a disproportionate inclusion of *MBNL1* exon 5 and *MBNL2* exons 5 and 8 occurs in cells with the (CTG)2600 repeat. As a consequence, a reduced quantity and imbalanced collection of splice variants of MBNL1 and MBNL2 accumulates in both the cytoplasm and the nucleus of DM1 myoblasts and myotubes. We thus propose that both the quantitative and qualitative changes in the intracellular partitioning of MBNL proteins are a pivotal cause of skeletal muscle problems in DM1, starting already in muscle progenitor cells.

## Introduction

Members of the Muscleblind-like (MBNL) protein family belong to a class of tissue-specific, developmentally programmed regulators of gene expression [1,2]. They control many aspects of RNA metabolism, such as alternative splicing and alternative polyadenylation, mRNA localization, translation and stability, and microRNA processing. In humans, like in other mammals, three *MBNL* isoforms, *MBNL1*, *MBNL2* and *MBNL3* are expressed. *MBNL1* and *MBNL2* are found ubiquitously, with *MBNL1* being more prominent in skeletal muscle and *MBNL2* relatively abundant in brain [3–5]. Expression of *MBNL3* is generally low in all tissues, with exception of liver and placenta [2,3,5,6].

*MBNL1-3* are highly homologous genes, of which the open reading frames are distributed over 9–10 exons, many of which are alternatively spliced [1,2]. Especially splicing of exons in the 3’ end of the primary *MBNL* transcripts is cell-type- and tissue-specific, and under developmental control [2,7–14]. Various combinations of exon inclusion and skipping events give rise to the production of a complex set of MBNL protein variants with different functional characteristics [1,2]. This process has been studied in detail predominantly for *MBNL1*. More specifically, inclusion of exon 5 (54 nts; nomenclature taken from Pascual et al. [1]) enhances nuclear localization of MBNL1, presence of exon 7 (36 nts) results in increased MBNL1 dimerization, while exon 3 (204 nts), almost always included, regulates MBNL1 RNA binding and splicing activities [8,10,11,13–16].

Aberrant alternative splicing of *MBNL1* and *MBNL2* is characteristic of the foetal splice pattern reported in patients with the severe neuromuscular disease myotonic dystrophy type 1 (DM1; OMIM#160900). In fact, functional down-regulation of *MBNL* isoforms is thought to be the actual cause of the pathological adult-to-foetal splice switch typical for this disease [2]. DM1 patients are characterized by the expression of an expanded (CTG)n repeat in the 3’ untranslated region of *DMPK* [17]. In unaffected individuals, the number of triplets in this gene varies between 5 and 37, but in patients with DM1 the repeat can expand to several thousand repeat units. Consequently, in tissues where the *DMPK* gene is expressed long pathological *DMPK* transcripts are formed. These RNAs remain retained in the cell nucleus where they form long hairpin structures that aberrantly sequester MBNL protein. This sequestration is associated with the formation of DMPK (CUG)n RNA-MBNL aggregates, which can be visualized as so-called foci by microscopy [18]. Also other effects on the intracellular partitioning of MBNL protein may occur. In turn, these processes may have widespread effects on muscle integrity and functioning, typical for the problems seen in DM1 patients. For example, reduced MBNL1 expression in proliferating DM1 myoblasts impairs foci formation, whereas enhanced MBNL1 expression induces nuclear retention of (CUG)n-expanded transcripts [13,19]. Moreover, *Mbnl* knockout mouse models replicate splicing abnormalities and disease symptoms of patients with DM1 [3,4,6,20–22].

All in all, there is overwhelming evidence that the *MBNL* gene family plays a crucial role in the muscle phenotype in DM1. Unfortunately, we still know remarkably little about the temporal effects of presence of an expanded (CTG)n repeat on the expression of *MBNL* family members during skeletal muscle myogenesis. We therefore present here a detailed study on RNA and protein expression, alternative splice modes and subcellular localization of MBNL isoforms in proliferating and differentiating cells of a human muscle cell model for DM1. This unique cell panel includes isogenic control myoblasts from which the expanded (CTG)2600 mutation has been excised via CRISPR/Cas9-mediated gene editing [23]. We found that RNA expression levels of *MBNL1*, *MBNL2* and *MBNL3* were similar in cells with and without the expanded repeat and did not noticeably change during myogenesis. However, at the post-transcriptional level, presence of the (CTG)2600 repeat caused a significant shift in alternative splicing of both *MBNL1* and *MBNL2* primary transcripts. At the post-translational level, this RNA splicing imbalance resulted in lower intracellular concentrations and also altered splice variant compositions of the MBNL1 and MBNL2 protein populations. These changes were already apparent in muscle precursor cells, the proliferating myoblasts, and were accompanied by a sustained, reduction of MBNL protein during differentiation to myotubes. Our study thus demonstrates a disturbed *MBNL* expression in DM1 pathology, already early during myogenesis, before myotube formation.

## Materials and methods

### Cell culture

Immortalized human DM1 myoblasts (named DM11) carrying *DMPK* alleles with (CTG)13 and (CTG)2600 repeats and CRISPR/Cas9-edited isogenic clones lacking both repeats as described [23] were used in this study. These myoblasts were propagated in a 1:1 mix of Skeletal Muscle Cell Growth Medium (PromoCell, Heidelberg, Germany) and F-10 Nutrient mix (Gibco, Carlsbad, CA, USA), supplemented with 15% (v/v) Hyclone foetal bovine serum (GE Healthcare Life Sciences, Chalfont St. Giles, UK) and 1% glutamax (Gibco), on culture dishes coated with 0.1% gelatin (Sigma-Aldrich, St. Louis, MO, USA), at 7.5% CO_2_ and 37°C. Myogenic differentiation to myotubes was induced by growing the myoblasts to confluency and replacing the proliferation medium by differentiation medium consisting of DMEM supplemented with 1% glutamax, 10 μg/mL insulin (Sigma-Aldrich) and 100 μg/mL apo-transferrin (Sigma-Aldrich). These low-serum conditions were maintained for up to five days. Culture medium was changed every other day.

### RNA isolation, RT-PCR and RT-qPCR

RNA was isolated using the Aurum Total RNA Mini Kit (Bio-Rad, Hercules, CA). RNA yield and purity were verified by absorbance at 260/280 nm (NanoVUE spectrophotometer, GE Healthcare Life Sciences). RNA was reverse transcribed using the iScript cDNA Synthesis Kit (Bio-Rad). To measure *MBNL1* and *MBNL2* splice variants, semi-quantitative PCR was performed using Q5 High-Fidelity DNA Polymerase (NEB) and the primers listed in S1 Table. The PCR program involved initial denaturation at 98°C for 3 min, followed by 30 cycles at 98°C for 10 seconds, 69°C (*MBNL1*) or 72°C (*MBNL2*) for 30 seconds and 72°C for 30 seconds, and a final extension at 72°C for 10 minutes. PCR products were analysed on the QIAxcel Advanced capillary electrophoresis system (QIAGEN, Venlo, Netherlands) using the DNA High Resolution Kit, along with the 15–600 bp alignment marker and 25–500 bp size marker. QIAxcel ScreenGel Software (1.5.0.16; QIAGEN) was used to quantify the various splice products.

For quantitative RT-PCR (RT-qPCR), 3 μL ten-fold diluted cDNA preparation was mixed in a final volume of 10 μL containing 5 μL iQ SYBR Green Supermix (Bio-Rad) and 4 pmol of each primer (S1 Table). Samples were analysed using a CFX96 Real-time System (Bio-Rad). A melting curve was obtained for each sample in order to confirm single product amplification. cDNA samples from no template control (NTC) and no reverse transcriptase control (NRT) were included as negative controls. *GAPDH* and *HPRT1* were used as reference genes.

### RNA sequencing and analysis

RNA was isolated as described from proliferating myoblasts growing at 80% confluency. RNA-sequencing (RNA-seq) libraries were prepared using the Unstranded TruSeq mRNA kit (Illumina, San Diego, CA, USA) and ~40 million 100-bp paired end reads were obtained using the Hiseq4000 platform (Illumina), all performed by the Beijing Genomics Institute (BGI, Hong Kong). Clean reads were mapped to the hG38 genome assembly using HISAT v0.1.6-beta [24] and transcripts were reconstructed using StringTie v1.0.4 [25]. Integrative Genomics Viewer [26] was used in conjunction with rMATS v3.0.9 [27] for quantification of differential splicing. For quantification of total expression levels RSEM v1.2.12 [28] was used after merging reads for transcripts with an identical reference transcript with BowTie2 v2.2.5 [29]. To compare expression levels of different genes FPKM values were used. Raw RNA-seq data and downstream analyses were deposited in the Gene Expression Omnibus under the accession number GSE127296.

### Immunofluorescence assay

Myoblasts were cultured on 0.1% gelatin-coated coverslips and fixed the next day. For myotube cultures, myoblasts were cultured in 0.1% gelatin-coated IBIDI 8-wells (IBIDI) and fixed the next day or after five days of differentiation in 2% PFA in 0.1 M phosphate buffer, pH 7.4. After fixation, cells were washed three times with PBS and permeabilized with an acetone methanol mixture (1:1 w/v). Cells were incubated in 3% bovine serum albumin (BSA) (w/v) (Sigma-Aldrich) in PBS for 30 min at room temperature. After overnight incubation at 4°C with primary antibodies (S2 Table) diluted in 3% BSA in PBS, the samples were washed three times with PBS and incubated with goat-anti-mouse AF488 (Thermo Fisher Scientific, Boston, MA, USA) and 100 ng/mL DAPI (Sigma-Aldrich) in blocking buffer (3% BSA and 0.1% (w/v) glycine in PBS) for one hour at room temperature. After three PBS washes, samples were stored in PBS at 4°C until imaging. Myoblast samples were imaged using a Leica DMI6000B high-content microscope with a 63x objective. Per coverslip, at least 30 cells were examined using the same acquisition time. Myotube samples were imaged using a Zeiss LSM880 microscope (Plan-Apochromat 20x N/A 0.8). Analysis of the signal was performed with FIJI software using the same size region of interest (ROI), randomly placed in the nucleus or cytosol of a myotube or myoblast, using the DAPI signal as mask for the nucleus.

### Protein extraction, SDS-polyacrylamide gel electrophoresis and western blotting

After two PBS washes, whole cells were lysed in 2x Laemmli sample buffer, followed by boiling for 5 minutes at 95°C. Resulting protein mixtures, along with ProSieve Quadcolor protein marker (Lonza), were electrophoresed on 10% SDS polyacrylamide gels in SDS running buffer. Proteins were transferred to Immobilin PVDF membrane (GE healthcare, 0.45 μm pore size) and membranes were blocked for one hour with 3% BSA in PBS with 0.1% Tween-20 (PBST) and incubated with primary antibodies (S2 Table) diluted in blocking buffer overnight at 4°C. Membranes were washed three times in PBST and incubated with appropriate IRDeye secondary antibody (IRDeye 800 CW goat anti-rabbit or IRDeye 680 LT goat anti-mouse; LI-COR Biosciences, Lincoln, NE, USA) in PBST for one hour and washed three times before being scanned in 700 nm and 800 nm wavelength channels on the Odyssey Clx imaging system (LI-COR Biosciences). Densitometry was performed using Image studio version 5.0 software (LI-COR Biosciences).

### Subcellular fractionation

For subcellular fractionation, myoblasts were grown to 80% confluence, collected by trypsinisation and pelleted by centrifugation at 1,000xg for 5 min at 4°C. Pellets were washed twice with ice-cold PBS. The cell pellets were resuspended in ice-cold cell disruption buffer, 10 mM KCl, 1.5 mM MgCl_2_, 20 mM Tris-Cl (pH 7.5), 1 mM DTT [30], and incubated on ice for 10 minutes. Cells were lysed by membrane disruption in a chilled Dounce homogenizer (tight pestle, 0.025–0.076 mm; Wheaton for 15 strokes) after which Triton X-100 was added to a final concentration of 0.1%. The lysate was spun at 1,500xg for 5 min at 4°C. The supernatant (cytoplasmic fraction) and the pellet (nuclear fraction) were each diluted in 2x Laemmli sample buffer for later use in SDS-PAGE. Enrichment for cytoplasmic and nuclear content was verified by western blotting using E7 tubulin and lamin A/C antibodies (S2 Table). For subcellular fractionation of myotubes, five-day differentiated myotube cultures were used. To separate multinucleated myotubes from mononuclear cells still present in the culture, myotubes were selectively released from the substrate by mild trypsinization at room temperature for 2 minutes. The myotube-enriched cell population was fractionated and analyzed as described for myoblasts except that the cell pellet was incubated in cell disruption buffer for 5 minutes and 20 strokes were used.

### Statistical analysis

All experiments were performed in triplicate unless otherwise specified and representative results are shown. Statistical analysis was performed using Prism software (4.01; GraphPad, LaJolla, CA), using two-way ANOVA with α = 0.05. * p<0.05, ** p<0.01, and *** p<0.001. Error bars indicate standard errors of the mean (SEM).

## Results

### *MBNL1*, *MBNL2* and *MBNL3* RNA levels are unchanged after excision of the (CTG)2600 repeat

**Fig 1:**
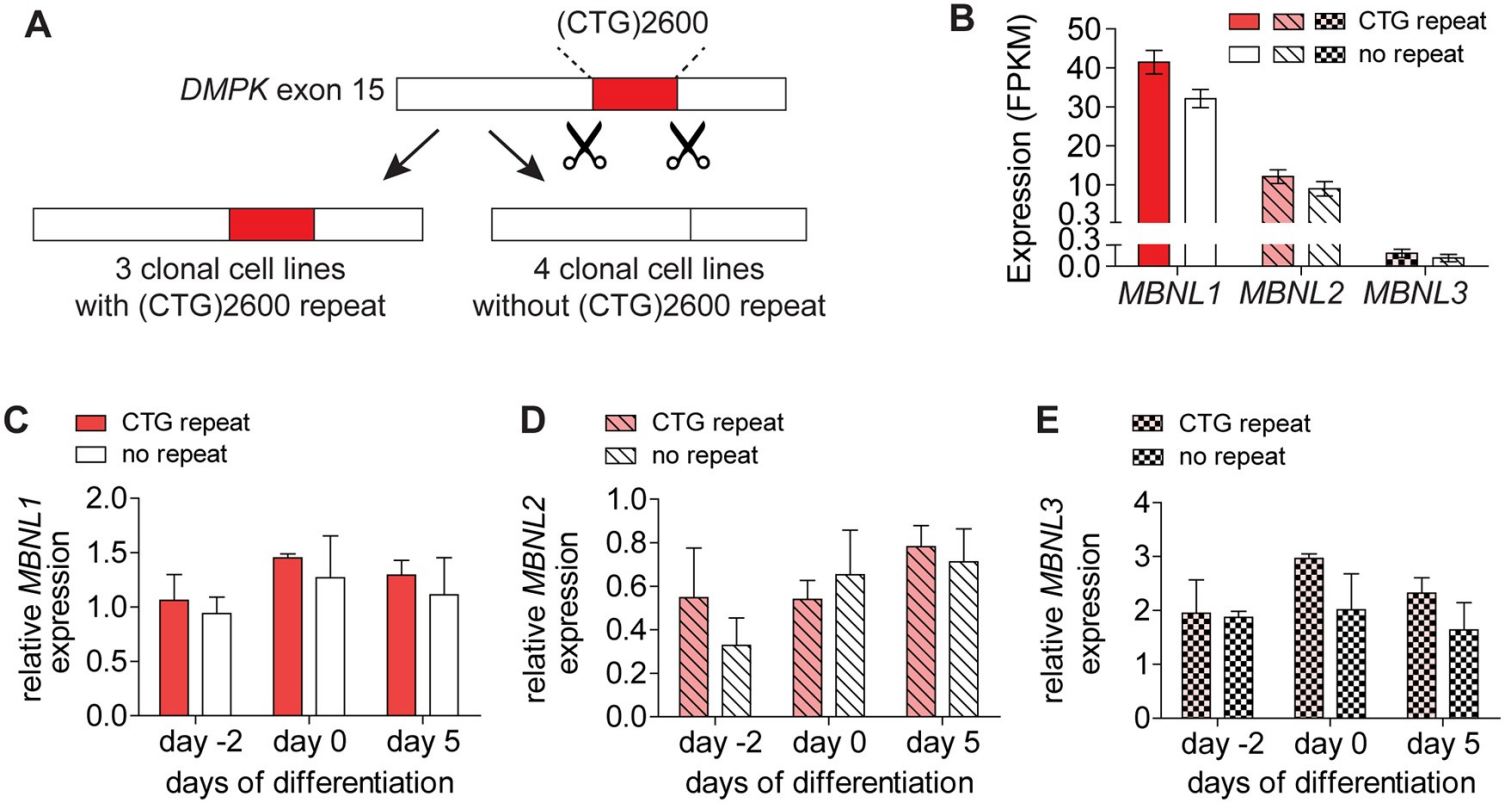
RNA expression of *MBNL* isoforms in myogenic cell cultures with and without expanded (CTG)2600 repeat. (A) Schematic outline of the CRISPR/Cas9 editing procedure by which the (CTG)2600 repeat was excised from the *DMPK* gene in the DM1 myoblast cell line named DM11 [23]. As a result, a panel of eight isogenic myoblast cell lines was obtained: four non-edited clonal cell lines with expanded (CTG)2600 repeat (the parental cell line and three independent clones) and four clonal lines from which the mutant repeat was removed. (B) *MBNL1*, *MBNL2* and *MBNL3* expression in proliferating myoblasts determined by RNA-seq. (C-E) Quantification of *MBNL1*, *MBNL2* and *MBNL3* RNA levels in proliferating myoblasts (day -2) and day 0 or 5 of myogenic differentiation measured by RT-qPCR. All bars show the mean values for the four cell lines with and without (CTG)2600 repeat expansion, all values are relative to the parental cell line.

To study RNA and protein expression of *MBNL* isoforms during myogenesis in DM1, we used the myoblast cell panel previously generated via gene editing in our lab (Fig 1A) [23]. The parental myoblast cell line from which the cell panel was derived carried 13 and 2600 CTG triplets in its two *DMPK* alleles [31]. Following the CRISPR/Cas9 editing procedure and careful cell cloning, we obtained four myoblast lines from which the mutant repeat was removed and four lines still carrying the (CTG)2600 repeat expansion (one of which being the parental cell line). Three of the four lines in the former group contained *DMPK* alleles lacking both the expanded and the normal repeat tract (the so-called ΔΔ lines), while one line still carried an unmodified (CTG)13 allele (line 13/Δ) [23]. This myoblast cell panel provides an elegant, isogenic study model to analyse effects of presence of a congenital DM1 repeat on endogenous *MBNL* expression.

First, to quantify *MBNL* expression, we performed RNA-seq analysis on proliferating myoblasts. Transcript levels of *MBNL1*, *MBNL2* and *MBNL3* in (CTG)2600 repeat-containing cells did not differ from those in cells from which the repeat expansion was removed (Fig 1B). When comparing the expression of the three individual *MBNL* isoforms, we found that *MBNL2* RNA abundance was around three times lower than that of *MBNL1*. *MBNL3* RNA was even much lower expressed, in fact around 250 times lower than *MBNL1* [32].

RT-qPCR analysis showed that *MBNL* expression was largely unresponsive to five days of myogenic differentiation *in vitro* (Fig 1C–1E). RNA abundance of all three genes in proliferating myoblasts (day -2), aligned myoblasts committed to cell fusion (day 0), and in differentiating myotube cultures (day 5) was similar between cell lines with and without (CTG)2600 repeat. This analysis further confirmed that expression of *MBNL1* and *MBNL2* was much higher than *MBNL3*. We decided to concentrate this study on *MBNL1* and *MBNL2*, the two most prominent *MBNL* isoforms in myogenic progenitor cells.

### Excessive exon inclusion of *MBNL1* exon 5 and *MBNL2* exons 5 and 8 occurs in cells with a (CTG)2600 repeat

**Fig 2:**
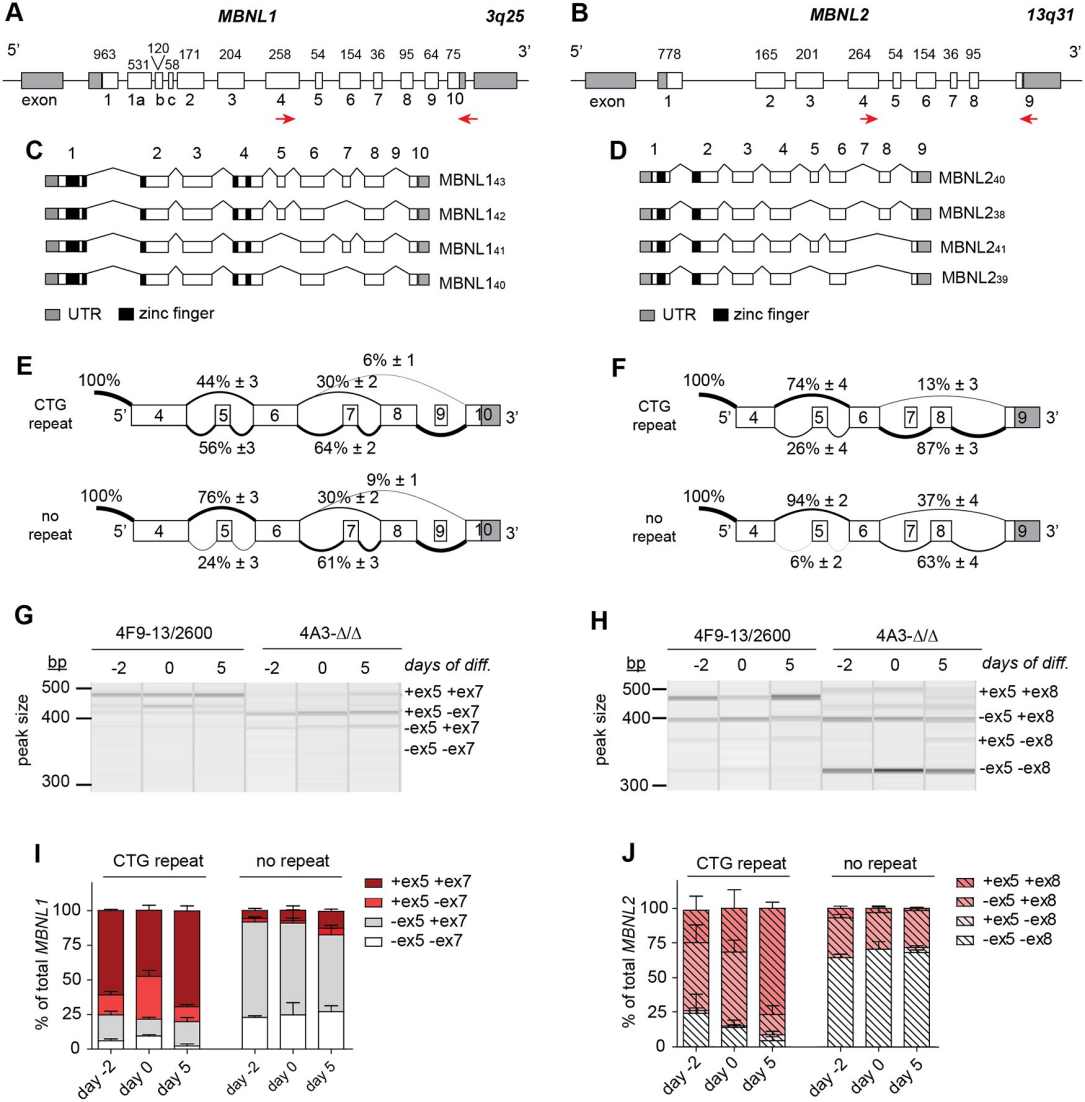
Alternative splicing of *MBNL1* and *MBNL2* during myogenic differentiation in vitro in presence and absence of an expanded (CTG)2600 repeat. (A, B) Gene structures of *MBNL1* and *MBNL2*, adapted from [1,11]. Boxes represent exons, numbered 1–10, connected by horizontal lines, the introns. Exon lengths are indicated in base pairs. Primers for RT-PCR are shown as red arrows. (C, D) The main alternative splice modes for *MBNL1* and *MBNL2* identified in this study and the molecular weights of the corresponding protein products, adapted from [1,11]. (E, F) Summary of RNA-seq data of the most prominent alternative splice modes of *MBNL1* and *MBNL2* in proliferating myoblasts with and without (CTG)2600 repeat (averaged data of four cell lines for each condition). Numerals indicate the percentage of reads spanning an upstream exon to a downstream exon. (G, H) Representative QIAxcel images of RT-PCR analyses of the exon 4 to exon 10/9 region in *MBNL1* and *MBNL2*, respectively, in proliferating myoblasts (-2) and at day 0 and 5 of myogenic differentiation. (I, J) Quantification of the RT-PCR data shown in (G, H).

Alternative splicing of *MBNL1* and *MBNL2* pre-mRNAs results in the production of a complex mix of at least a dozen possible splice variants for each isoform (see Fig 2A–2D for the *MBNL1* and *MBNL2* exon nomenclature used in this paper [1,8,9,12,14]. In adult DM1 muscle, abnormal inclusion of *MBNL1* exon 5 (54 nts) is an accepted hallmark for the foetal splice pattern characteristic for the disease [8,9,12]. Abnormal splicing of *MBNL2* in DM1 has not been studied in detail yet, but it has been reported that also for this isoform the conserved exon 5 (54 nts) is overrepresented in mature transcripts [8,10].

We have analysed *MBNL1* and *MBNL2* splicing in proliferating myoblasts using RNA-seq and focussed on the main alternatively spliced regions, encompassing *MBNL1* exons 5–9 and *MBNL2* exons 5–8 (Fig 2A–2D) [1,14]. For *MBNL1*, we identified manifest alternative use of exons 5, 7 and 8, while exons 6 and 9 were nearly always constitutively included and excluded, respectively. In the case of *MBNL2*, exons 5 and 8 were alternatively spliced, while exons 6 and 7 were nearly always included and excluded, respectively. Exons 1–4, including alternative exon 3, were present in essentially all *MBNL1* and *MBNL2* transcripts in the samples examined.

When comparing myoblasts carrying the (CTG)2600 repeat with cells from which the repeat had been recently removed, we found that *MBNL1* exon 5 was preferentially included in cells with the repeat (56% vs 24%; p<0.001; FDR = 0.0001) (Fig 2E). No significant differences were seen for *MBNL1* exon 7 (64% vs 61%) nor exon 8 (94% vs 91%). For *MBNL2*, we measured increased inclusion of exon 5 (26% vs 6%; p<0.001; FDR = 0.007) and exon 8 (87% vs 63%; p<0.001; FDR = 0.0005) in myoblasts with a (CTG)2600 repeat (Fig 2F).

We subsequently performed semi-quantitative RT-PCR across *MBNL* exons 5 to 9/8 on RNA isolated from proliferating and differentiating myoblasts to verify the RNA-seq data and to investigate the combinatorial use of multiple alternative splice events during myogenesis. We could confirm that in proliferating myoblasts containing the (CTG)2600 expansion the majority of *MBNL1* transcripts included exon 5, unlike in myoblasts without the expanded repeat (75% vs 8%; Fig 2G and 2I). We assume that the slightly deviating ratios obtained by RT-PCR and RNA-seq analysis are due to fundamental technical dissimilarities between the two methods. Inclusion of *MBNL1* exon 7 was indeed independent of expanded repeat presence (80% vs 75%). When we looked more specifically at combinatorial use of exons 5 and 7, we found that the +ex5+ex7 and +ex5-ex7 *MBNL1* variants, encoding MBNL1_43_ and MBNL1_42_ proteins respectively, were around nine-fold higher expressed in cells carrying an expanded repeat than in cells without repeat. In contrast, the -ex5+ex7 and -ex5-ex7 variants, corresponding to the smaller MBNL1_41_ and MBNL1_40_ proteins respectively, were four-fold less abundant in cells with repeat. Of note, despite the fact that alternative splicing and myogenesis are intrinsically related processes, the *MBNL1* splice pattern did not undergo an overt change upon the onset of differentiation or the next five days of further myoblast-to-myotube differentiation *in vitro*, irrespective of repeat presence.

RT-PCR analysis of the *MBNL2* splice pattern confirmed that proliferating myoblasts containing the (CTG)2600 expansion showed a higher degree of exon 5 and exon 8 inclusion than those without (25% vs 7%, and 74% vs 36%, respectively) (Fig 2H and 2J). We observed that the +ex5+ex8 and -ex5+ex8 *MBNL2* mRNA variants, encoding MBNL2_40_ and MBNL2_38_ proteins respectively, were both around two-fold higher expressed, whereas the -ex5-ex8 variant, encoding MBNL2_39_ protein was around three-fold reduced in (CTG)2600 containing myoblasts. The +ex5-ex8 mRNA variant, encoding MBNL2_41_ protein, could barely be detected at all. Remarkably, *MBNL2* exon 5 and exon 8 inclusion increased significantly during myogenic differentiation, but only in cells with the (CTG)2600 repeat. This phenomenon seems a distinct feature of *MBNL2* expression, as it was not observed for *MBNL1*.

All data combined, we conclude that alternative *MBNL* splicing is noticeably perturbed during the proliferative phase of DM1 myoblast cell formation and involves increased inclusion of *MBNL1* exon 5 and *MBNL2* exons 5 and 8. Upon induction of myogenic differentiation, this situation is maintained during the subsequent phase of quiescence and myoblast fusion into myotubes, whereby a specific transition occurred between *MBNL2* +ex5+ex8 (MBNL2_40_) and -ex5+ex8 (MBNL2_38_) variants.

### (CTG)2600 repeat-induced alternative splicing results in reduced MBNL1 and MBNL2 levels and altered protein composition

We next examined whether the abnormally spliced, yet similar total levels of *MBNL1* and *MBNL2* mRNAs would significantly alter MBNL protein expression in cells with a CTG(2600) repeat compared to cells lacking the repeat. To this end, whole-cell protein lysates of proliferating myoblast cultures and of cultures harvested at different time points after initiation of differentiation were prepared and used in western blotting. The MBNL1 antibody MB1a that we used for detection was directed against an epitope encoded by exon 3 [33], present in essentially all MBNL1 variants in our cells.

**Fig 3:**
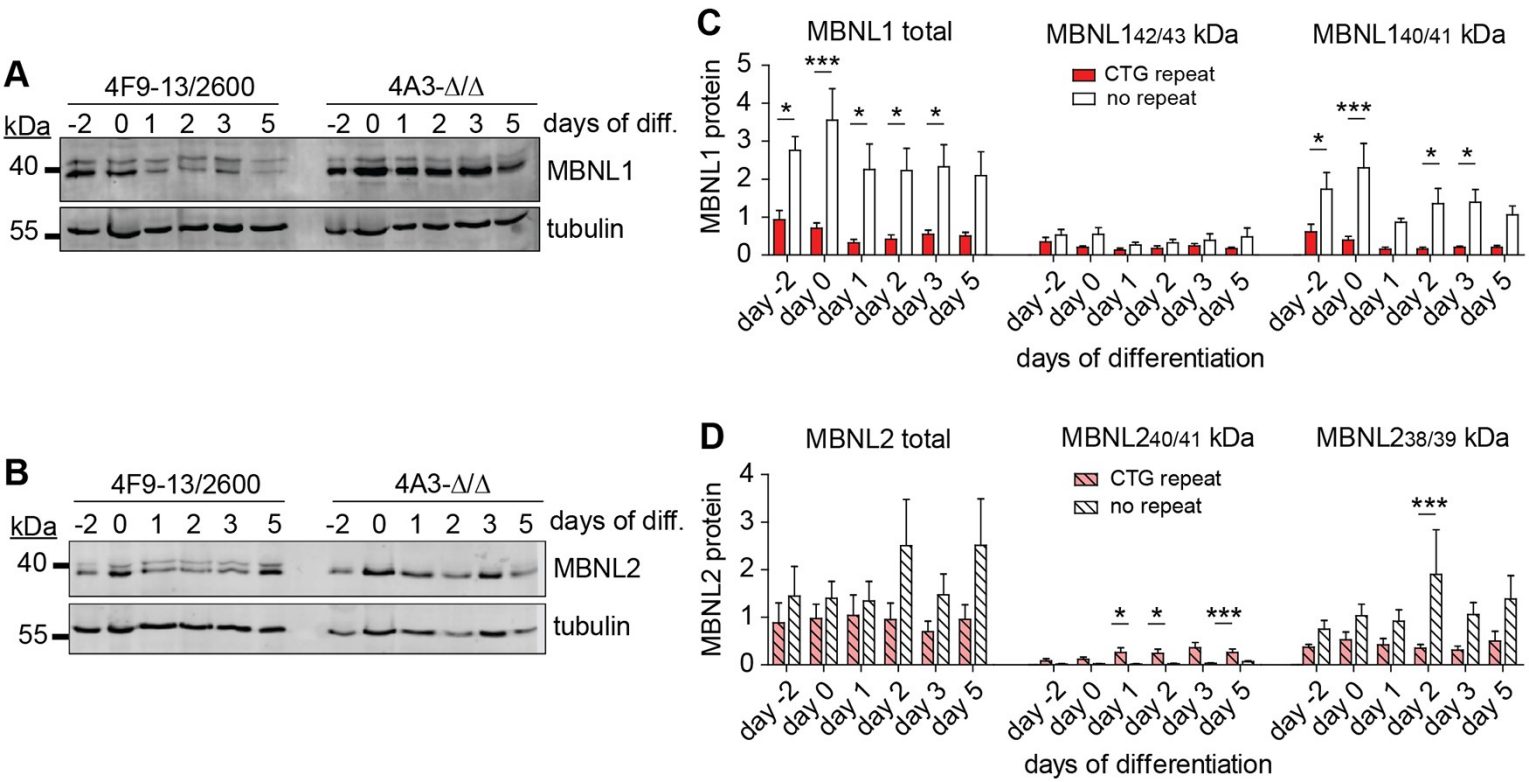
MBNL1 and MBNL2 expression in proliferating and differentiating myogenic cells with and without (CTG)2600 repeat. (A,B) Representative western blots for MBNL1 and MBNL2 from cell lines with (4F9-13/2600) and without (4A3-ΔΔ) the (CTG)2600 repeat. (C,D) Quantification of MBNL1 and MBNL2 expression, including specific variants, in arbitrary units (a.u.) after normalization to β-tubulin expression. Bars summarize data from two cell lines with and two cell lines without (CTG)2600 repeat. Each cell line was measured three times, in independent experiments.

From the inferred amino acid (aa) sequences, we predicted that exon 5 or exon 7 inclusion would contribute an extra 2.0 kDa (18 aa) or 1.2 kDa (12 aa), respectively to the total molecular weight of the MBNL1 variants. The blots showed a complex collection of MBNL1 signals with migration behaviour around the 40 kDa marker protein, as expected (Fig 3A). Two bands were most prominent, irrespective of the presence of an expanded repeat in the cells. Taking our RT-PCR results and the available literature data [11,12,33] into account, we assumed that the top signal represented mainly MBNL1_43_ (+ex5+ex7) cosegregating with MNL1_42_ (+ex5-ex7), while in the bottom signal MBNL1_41_ (-ex5+ex7) and MBNL1_40_ (-ex5-ex7) migrated together. Total MBNL1 levels were three to four-fold lower in proliferating myoblasts with a (CTG)2600 repeat than in cells without the repeat (Fig 3C). This difference in expression was mainly caused by a reduction in the MBNL1_40/41_ (-ex5±ex7) variants (bottom signal), the dominant proteins in cells lacking the repeat (approximately 75% of total MBNL1). More specifically, during five days of differentiation, the total MBNL1 level, again mainly the MBNL1_40/41_ variants, decreased around two-fold, particularly in cultures with a (CTG)2600 repeat.

Also for MBNL2, multiple signals were detected, using antibody MB2A directed at the N-terminus shared by all MBNL2 variants [33] (Fig 3B). Inclusion of exon 5 would contribute an extra 2.0 kDa (18 aa) to MBNL2, while exon 8 inclusion initiates a shift in the open reading frame resulting in a novel C-terminus and a reduction in molecular weight of 1.2 kDa. The top signal, most prominently observed in cells with the (CTG)2600 repeat probably reflected MBNL2_40_ (+ex5+ex8), cosegregating with MBNL2_41_ from the barely expressed +ex5-ex8 splice variant. We assumed that the bottom signal represented a mix of MBNL2_39_ (-ex5-ex8) and MBNL2_38_ (-ex5+ex8). Total MBNL2 levels were not significantly lower in cells with a (CTG)2600 repeat than in cells without the repeat (Fig 3D). However, in cells containing the (CTG)2600 repeat, the MBNL2_40/41_ (+ex5±ex8) variants were significantly more abundant under differentiation conditions; in fact, these MBNL2 variants were essentially absent in cells from which the repeat expansion had been removed. In contrast to MBNL1, total MBNL2 level remained relatively constant during myogenic differentiation.

In sum, the total level and protein composition of both MBNL1 and MBNL2 is altered in the presence of the expanded (CTG)2600 repeat. During myogenic differentiation, MBNL1, but not MBNL2, levels decreased slightly, particularly in cells with the repeat.

### MBNL1 and MBNL2 are reduced in the nucleus and in the cytoplasm in DM1 myoblasts and myotubes

We wondered about the effects of lower protein expression and altered protein composition on MBNL localization in the cell. It has been reported that inclusion of *MBNL1* exon 5 is implicated in the nuclear localization of MBNL1 [10,11,13]. Moreover, the exon 5-encoded amino acid stretch is highly conserved in *MBNL2*. We employed immunofluorescent microscopy using the same antibodies as for western blotting, and examined the subcellular distribution of MBNL1 and MBNL2 in myoblasts with and without (CTG)2600 repeat.

**Fig 4:**
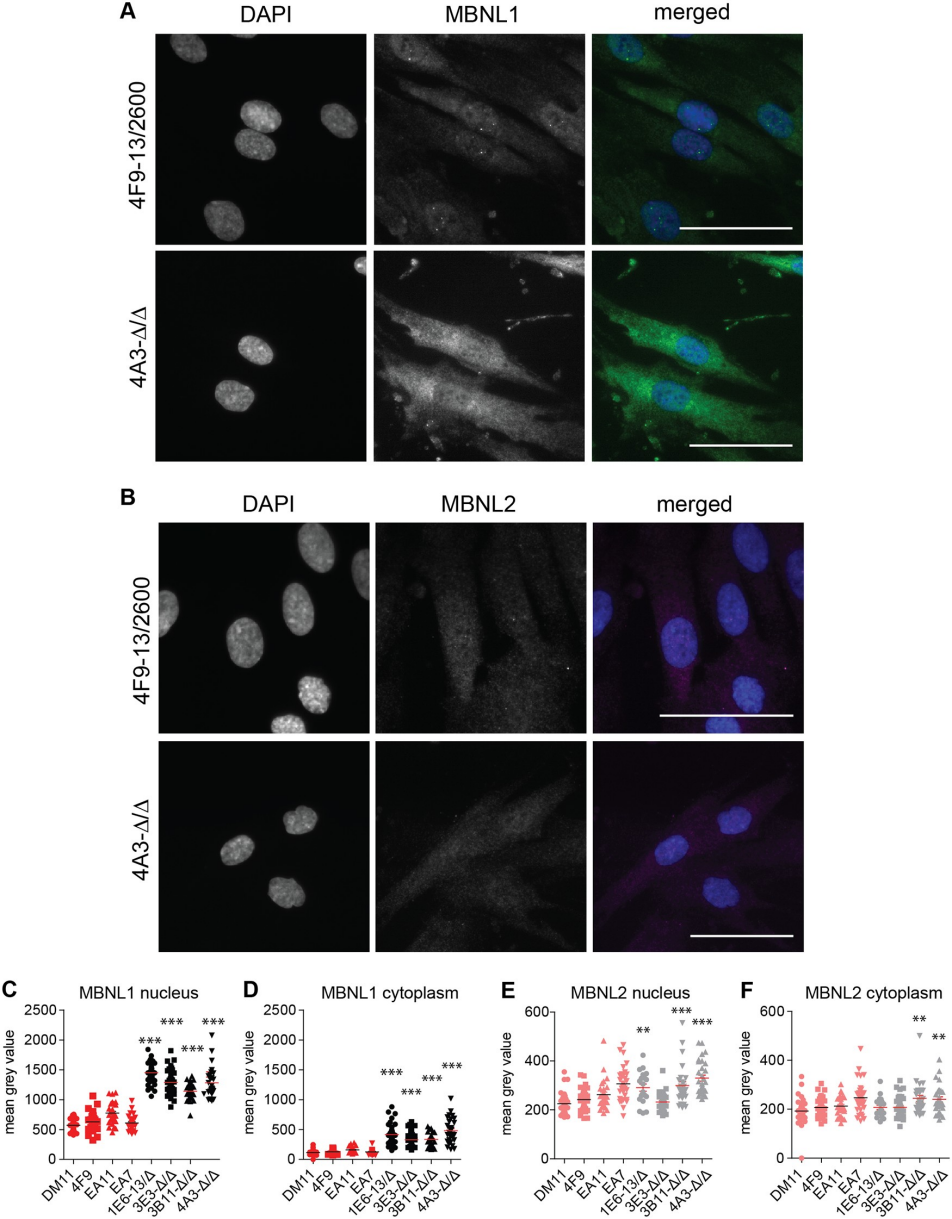
Subcellular localization of MBNL1 and MBNL2 in myoblasts with and without (CTG)2600 repeat. (A, B) Immunofluorescent localization of MBNL1 and MBNL2 in proliferating myoblasts with (4F9) and without (4A3) the (CTG)2600 repeat expansion. Merged images show DAPI in blue, MBNL1 in green and MBNL2 in magenta. Scale bars are 50 μm. (C-F) Quantification of MBNL1 and MBNL2 levels in the nucleus and the cytoplasm in all eight cell lines. Each symbol represents the average intensity in one myoblast.

The typical DM1 foci in the nuclei of cells with the expanded (CTG)n repeat contained MBNL1 as well as MBNL2 (Fig 4A and 4B; on average around 3 foci per nucleus). Notably, only a fraction of MBNL1 and MBNL2 was contained within these foci and these typical intense signals were not present in cells lacking the repeat [23]. In fact, we always observed a diffuse, homogenous distribution of MBNL1 and MBNL2 in both the cytoplasm and the nucleus. Careful quantification of MBNL1 staining in individual cells through advanced image analysis showed that the average MBNL protein content was three to four-fold higher in the nucleus than in the cytoplasm in all cell lines (Fig 4C and 4D). Notably, up to five-fold differences in intensity were measured between cells of one clonal population, illustrating a high cell-to-cell variability. More importantly, when comparing the staining intensities between the two cell populations, we consistently measured a two to three-fold higher MBNL1 intensity in both compartments in cells lacking the (CTG)2600 repeat (Fig 4C and 4D). In contrast, MBNL2 staining intensities were essentially equal in the nucleus and the cytoplasm, while only some clones without the (CTG)2600 repeat displayed a significantly higher expression in both cellular compartments than cell lines with the expanded repeat (Fig 4E and 4F).

Since immunofluorescence detection *in situ* using antibodies MB1a and MB2a did not allow for discrimination between MBNL variants, we performed subcellular fractionation of proliferating myoblasts followed by western blotting to identify the MBNL1 and MBNL2 variants in the cytoplasmic and nuclear-enriched fractions. As expected, the MBNL1_42/43_ and MBNL2_40/41_ variants, corresponding to exon 5 inclusion, were enriched in the nuclear fractions, particularly in cells with the expanded (CTG)2600 repeat (S1A and S1B Fig).

**Fig 5:**
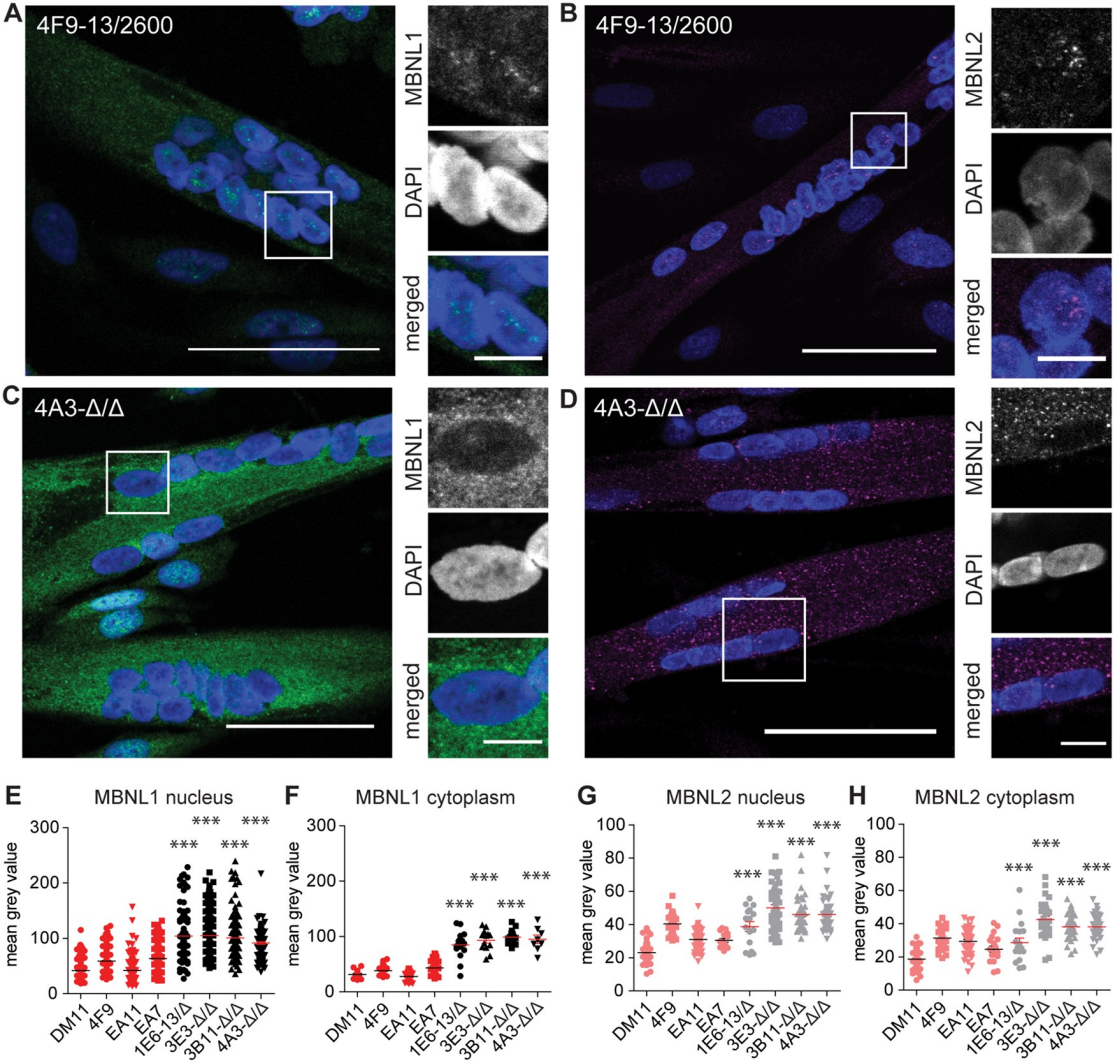
Subcellular distribution of MBNL1 and MBNL2 in five-day differentiated myotubes with and without (CTG)2600 repeat. (A-D) Immunofluorescent localization of MBNL1 and MBNL2 in myotubes with (4F9) and without (4A3) the (CTG)2600 repeat expansion, after five days in differentiation medium. Images show DAPI in blue, MBNL1 in green and MBNL2 in magenta. Bars are 50 μm in large images and 10 μm in insets. (E-H) Quantification of MBNL1 and MBNL2 levels in the nucleus and the cytoplasm in all eight cell lines. Each symbol represents the average intensity in one multinucleated myotube.

The subcellular localization of MBNL was also determined in five-day old multinucleated myotubes. For both MBNL1 and MBNL2, next to the nuclear foci in (CTG)2600 repeat-containing cells, we observed a diffuse granular staining in the nucleus and in the cytoplasm (Fig 5A–5D). Excision of the (CTG)2600 repeat resulted in a two to three-fold higher MBNL1 intensity in the nuclear and cytoplasmic compartment, respectively, and a 1.5-fold higher MBNL2 intensity in both compartments (Fig 5E–5H). More importantly, in contrast to the localization in myoblasts, we measured on average in myotubes an essentially equal distribution of MBNL1 and MBNL2 throughout the cell, irrespective of repeat presence. It should be noted however, that we regularly observed myotubes containing nuclei with highly variable MBNL1 and MBNL2 intensities. Subcellular fractionation followed by western blotting confirmed that also in myotubes MBNL1_42/43_ and MBNL2_40/41_ variants were more abundant in the nuclear fractions, particularly in cells with the expanded (CTG)2600 repeat (S1C and S1D Fig).

## Discussion

As important cell type-specific regulators of RNA processing, *MBNL* family members must be spatiotemporally expressed with high accuracy during development. Functional loss or change in the composition of the cellular MBNL isoform population is thought to be, at least in part, responsible for the spliceopathy typically seen in DM1 [34]. In the present study, we examined *MBNL* expression, with a focus on *MBNL1* and *MBNL2*, in a well-controlled human cell model for DM1 with normal expression of an expanded repeat. Our unique isogenic muscle cell panel, comprising several independent cell lines with and without a congenital DM-type mutation, enabled us to study repeat effects without possible confounding influences of genetic background or other forms of interpatient variability. Using independent methods for the identification and quantification of RNA and protein, we investigated *MBNL* expression in detail in proliferating myoblasts and in differentiating myotubes. Earlier, we and others had already shown that (CTG)n repeat excision in DM1 cells restores the normal cellular phenotype [23,35,36]. With regard to the progressive muscle phenotype in DM1, we conclude here that (CTG)n-repeat toxicity perturbs *MBNL* expression as early as in myoblasts, the muscle progenitor cells.

RNA-seq and RT-PCR analyses demonstrated that removal of the (CTG)2600 repeat did not alter RNA expression levels of any of the three *MBNL* isoforms in our cell model. This observation matches findings from a comparative RT-qPCR study on muscle biopsies from a cohort of DM1 patients and unaffected individuals [37] and a recent large transcriptomics study on quadriceps samples from patients and controls [38]. Interestingly, the latter study reported a slight increase in *MBNL1* expression, but not *MBNL2* and *MBNL3*, in tibialis muscle from DM1 patients compared to controls, indicating that expression levels can vary in different muscle tissues [38]. We also observed that *MBNL* RNA levels did not overtly change during myoblast alignment and myotube formation during five days *in vitro*. All in all, these RNA quantifications indicate that changes in *MBNL* protein expression in early myogenesis in DM1 must be predominantly the result of (CTG)n repeat-mediated effects at the posttranscriptional level.

Probably the main level of posttranscriptional regulation of *MBNL* is alternative splicing. Alternative splicing of *MBNL1* and, to a limited extent, *MBNL2* has been studied by a number of laboratories, but only rarely under well-controlled conditions of normal expression of an expanded DM1 repeat [2,7–14]. The current literature is confusing with regard to *MBNL* gene structure, but using the nomenclature summarized in Fig 2 [1], we could confirm alternative splicing of *MBNL1* exons 3 (204 nts), 5 (54 nts), 7 (36 nts) and 8 (95 nts) in muscle cells. Of these, we basically only found a significant increase of exon 5 inclusion in DM1 cells. We did not detect increased inclusion of exon 7, which was reported in DM1 brain [9]. The two alternatively spliced *MBNL2* exons, exon 5 (54 nts) and exon 8 (95 nts), were both preferentially included in DM1 cells. It is important to note that we mainly detected a shift in splice variant composition of *MBNL1* and *MBNL2* and did not identify significant expression of uncommon or novel splice variants.

When comparing the data on RNA and protein expression from either cells with or without (CTG)2600 repeat, we did not observe a quantitative one-to-one correlation between alterations in *MBNL* mRNA and protein splice variant composition, indicating additional regulatory mechanisms after the *MBNL* mRNA has been made. The most likely explanation for the difference in the regulation of *MBNL* mRNA and protein abundance are variations in mRNA translation efficiency and protein stability [39,40]. The latter may be related to differences in multimerization behaviour and subcellular localization between splice variants. Detailed analysis of MBNL splice variant synthesis rate and half-life, which is beyond the scope of this paper, is needed to clarify this issue.

We found that MBNL1 protein, in particular MBNL1_41_ (-ex5+ex7) was significantly reduced in myoblasts with repeat and even stronger in myotubes. As a result, MBNL1_43_ (+ex5+ex7) was overrepresented in these cells. Total MBNL2 protein level did not significantly change upon repeat expansion, but MBNL2_40_ (+ex5+ex8) increased at the expense of MBNL2_39_ (-ex5-ex8), particularly in myotubes. We therefore conclude that the dysfunction of *MBNL1* and *MBNL2* in DM1 consists of a moderate (two to five-fold) reduction in protein expression, combined with a modified splice variant content, i.e. overrepresentation of predominantly +ex5 variants. Effects of MBNL protein loss in DM1 have been studied in detail *in vivo* in *Mbnl* knockout mice, which showed that *Mbnl1* and *Mbnl2* have distinct functions, predominantly in muscle and brain respectively, but also that the two isoforms can partially compensate for each other’s absence [3,4,21,22]. Effects of altered splice variant compositions, but normal total levels, of MBNL1 and MBNL2 protein has not been studied yet, to our best knowledge.

Alternative splicing determines MBNL1 and MBNL2 localization in the cell, next to effects on RNA and protein binding, dimerization behaviour and splicing activity [8,10,11,13–16]. In particular, inclusion of the highly conserved exon 5 has been shown to be responsible for nuclear localization of MBNL1 [1,2]. Despite preferential inclusion of exon 5 in DM1 cells and accumulation of this variant in the nucleus, we consistently measured a reduced MBNL1 and, to a much lesser extent, MBNL2 expression in both the nucleus and the cytoplasm. The decrease was most prominent in myoblasts carrying the (CTG)2600 repeat, but also evident in the corresponding myotubes. A reduced staining in the nucleoplasm of cortical neurons from DM1 patients has been reported earlier [41]. In our muscle cells, irrespective of (CTG)2600 presence, MBNL1 concentration was highest in the nucleus, while MBNL2 was more or less equally present in nucleus and cytoplasm. Importantly, as expected, MBNL staining was concentrated in foci, but these signals were only a fraction of the total MBNL staining in the nuclei. The difference in foci number and the relatively large variations in MBNL protein staining in nuclei and cytoplasm between cells, even in one clonal population, could reflect transcriptional bursts of *DMPK*, *MBNL1* and *MBNL2* [42]. Recently, Wang et al. reported on the involvement of ubiquitination in the subcellular distribution of MBNL1 in neurons [43], but we have not been able to identify this type of post-translational modification for MBNL1 in our model system.

An important question that remains is how pathogenic *DMPK* (CUG)n transcripts trigger the prominent changes in the posttranscriptional regulation of *MBNL*. Presumably, expanded (CUG)n RNA-MBNL binding and subsequent formation of foci is the initiating event of the toxic cascade. After all, the use of antisense oligonucleotides and small molecules that specifically block this interaction has demonstrated that repeat RNA toxicity effects are reversible and can be neutralized [44,45]. We assume therefore that (CUG)n RNA-MBNL binding induces a mechanism that directly or indirectly perturbs *MBNL* splicing. Whether the number of MBNL1 and MBNL2 molecules that can be sequestered in foci is sufficient to significantly reduce the active MBNL concentration in the cell is questionable. However, MBNL1 is known to stimulate its own exon 5 skipping [46], so an initial small reduction in protein level could in fact catalyse more robust, sustainable and persistent changes in *MBNL1* splicing and protein variant composition. Alternatively, there might be specific signalling mechanisms that are activated following (CUG)n RNA-MBNL complex formation and that feed into splicing regulation, but these pathways have not been identified thus far.

In conclusion, we have demonstrated that dysregulation of *MBNL1* and *MBNL2* in DM1 is independent of differentiation cues, already occurring in myoblasts, the *in vitro* equivalent of muscle progenitor cells to the satellite cells *in vivo*. Disrupted *MBNL* expression pertains to protein level and splice variant composition throughout the entire cell. To obtain further insight in the exact sequence of events during muscle development, it is important to study *MBNL* expression even earlier during embryogenesis, right at the first appearance of toxic expanded (CUG)n RNA.

## Supporting information

S1 Fig Subcellular fractionation of proliferating myoblasts and differentiating myotubes to identify MBNL1 and MBNL2 variants in cytoplasm and nucleus. Western blot analysis of (A, C) MBNL1 and (B, D) MBNL2 in cytoplasmic (cyto) and nuclear-enriched (nucl) fractions of (A, B) proliferating myoblasts and (C, D) five-day old myotubes with and without (CTG)n repeat expansion. Lamin A/C and tubulin served as nuclear and cytoplasmic marker, respectively, to demonstrate enrichment of both fractions. Molecular weights are indicated in kDa. Note that the blots generally show four distinct signals for both MBNL1 and MBNL2, instead of the two usually detected, which represent comigrating protein variants (e.g. Fig 3). MBNL1_42/43_ and MBNL2_40/41_ variants (the top two bands in both panels), corresponding to exon 5 inclusion, were enriched in the nuclear fractions, particularly from cells with the expanded (CTG)2600 repeat.
(TIF)

S1 Table Primers used in this study. (PDF)

S2 Table Antibodies used in this study. (PDF)
