# Supplementary figures and images for: (CTG)n repeat-mediated dysregulation of MBNL1 and MBNL2 expression during myogenesis in DM1 occurs already at the myoblast stage
Source: PLoS One. 2019 May 22;14(5):e0217317. doi: 10.1371/journal.pone.0217317 (PMC6530876; doi:10.1371/journal.pone.0217317)

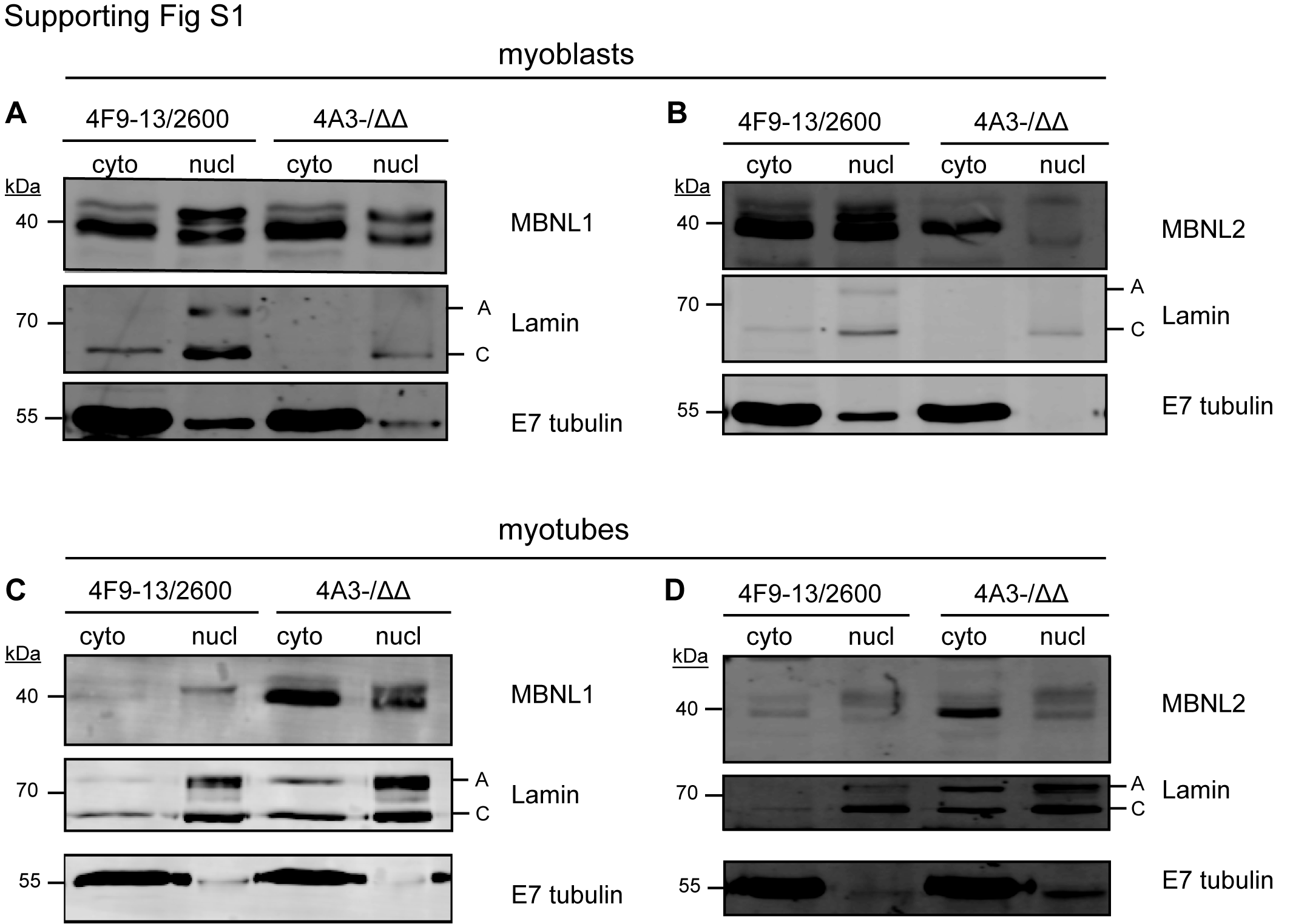

Supplement: S1 Fig — Western blot analysis of (A, C) MBNL1 and (B, D) MBNL2 in cytoplasmic (cyto) and nuclear-enriched (nucl) fractions of (A, B) proliferating myoblasts and (C, D) five-day old myotubes with and without (CTG)n repeat expansion. Lamin A/C and tubulin served as nuclear and cytoplasmic marker, respectively, to demonstrate enrichment of both fractions. Molecular weights are indicated in kDa. Note that the blots generally show four distinct signals for both MBNL1 and MBNL2, instead of the two usually detected, which represent comigrating protein variants (e.g. Fig 3). MBNL142/43 and MBNL240/41 variants (the top two bands in both panels), corresponding to exon 5 inclusion, were enriched in the nuclear fractions, particularly from cells with the expanded (CTG)2600 repeat. (TIF) [file pone.0217317.s001.tif]
