# Supplementary material for: (CTG)n repeat-mediated dysregulation of MBNL1 and MBNL2 expression during myogenesis in DM1 occurs already at the myoblast stage
Source: PLoS One. 2019 May 22;14(5):e0217317. doi: 10.1371/journal.pone.0217317 (PMC6530876; doi:10.1371/journal.pone.0217317)
Supplement: S1 Table — (PDF) [file pone.0217317.s002.pdf]

Supporting Information Table S1 – Primers used in this study

André et al., (CTG)n repeat-mediated dysregulation of MBNL1 and MBNL2 expression during myogenesis in DM1 occurs already at the myoblast stage.

**Gene**	**Purpose**[a]	**Sequence**[b]	**Product size(s) (bp)**
MBNL1	RT-qPCR	Ex1_Fw: GCTGTTAGTGTCACACCAATTCG<br>Ex1_Rv: AGGCGATTACTCGTCCATTTTC	154
MBNL1	Alternative splicing	Ex4_Fw: GGCTGCCCAATACCAGGTCA<br>Ex10_Rv: CTTGTGGCTAGTCAGATGTTCGG	470/434/416/380
MBNL2	RT-qPCR	Ex1_Fw: TCAAAGAGGAACATGCTCACG<br>Ex2_Rv: AACGGCCCTTTAGGGAATCAA	116
MBNL2	Alternative splicing	Ex4_Fw: TTTTCACCCTCCTGCACACT<br>Ex9_Rv: GTTATTCTCAATGCAGATTCTTGGC	470/416/375/321
MBNL3	RT-qPCR	Ex7_Fw: CAATACTGTGCATGGCACCCG<br>Ex8_Rv: GGTGGCAGGTGTTGTTGCTG	92
GAPDH	RT-qPCR	Fw: CCCGCTTCGCTCTCTGCTCC<br>Rv: CCTTCCCCATGGTGTCTGAGCG	94
HPRT1	RT-qPCR	Fw: TGACACTGGCAAAACAATGCA<br>Rv: GGTCCTTTTCACCAGCAAGCT	94

[a] RT-qPCR = quantitative RT-PCR to measure transcript levels; Alternative splicing = semi-quantitative RT-PCR to identify splice variants

[b] Fw = forward primer, Rv = reverse primer; all in 5’ -> 3’ direction
