# Supplementary material for: (CTG)n repeat-mediated dysregulation of MBNL1 and MBNL2 expression during myogenesis in DM1 occurs already at the myoblast stage
Source: PLoS One. 2019 May 22;14(5):e0217317. doi: 10.1371/journal.pone.0217317 (PMC6530876; doi:10.1371/journal.pone.0217317)
Supplement: S2 Table — (PDF) [file pone.0217317.s003.pdf]

Supporting Information Table S2 – Antibodies used in this study

André et al., (CTG)n repeat-mediated dysregulation of MBNL1 and MBNL2 expression during myogenesis in DM1 occurs already at the myoblast stage.

Protein	Company and antibody description	Dilution
MBNL1	Developmental Studies Hybridoma Bank, MB1a (4A8), recognizing the linker region between the two zinc finger domains in MBNL1 encoded by exon 3 [33]. Exon 3 was present in >97% of the MBNL1 mRNAs in our cells (data not shown). Mouse monoclonal.	1:100
MBNL2	Developmental Studies Hybridoma Bank, MB2a (3B4), recognizing the N-terminal sequence shared by all MBNL2 splice variants [33]. Mouse monoclonal.	1:100
β-Tubulin	Developmental Studies Hybridoma Bank (E7). Mouse monoclonal.	1:5000
MHC	Developmental Studies Hybridoma Bank (MF-20). Mouse monoclonal.	1:100
Lamin A/C	Abcam (ab40567). Mouse monoclonal.	1:500
